# Characterization of Glycoprotein 5-Specific Response in Pigs Vaccinated with Modified Live Porcine Reproductive and Respiratory Syndrome Virus Vaccine Derived from Two Different Lineages

**DOI:** 10.3390/vaccines13030247

**Published:** 2025-02-27

**Authors:** Jing Huang, Venkatramana D. Krishna, Igor A. D. Paploski, Kimberly VanderWaal, Declan C. Schroeder, Maxim C.-J. Cheeran

**Affiliations:** Department of Veterinary Population Medicine, College of Veterinary Medicine, University of Minnesota, St. Paul, MN 55108, USA; huan2167@umn.edu (J.H.); vdivanak@umn.edu (V.D.K.); ipaplosk@umn.edu (I.A.D.P.); kvw@umn.edu (K.V.); dcschroe@umn.edu (D.C.S.)

**Keywords:** porcine reproductive and respiratory syndrome virus, glycoprotein 5, neutralization, modified live virus vaccine, epitope

## Abstract

Background/Objectives: Porcine reproductive and respiratory syndrome virus (PRRSV) is classified into various lineages based on the phylogenetic variation of *orf5*, which encodes a major surface glycoprotein GP5 containing both neutralizing and non-neutralizing linear epitopes. Several positively selected sites have been identified on the GP5 ectodomain, indicating host immune pressure on these sites. This present study aimed to investigate the kinetics of antibody responses to GP5 and to map the epitope-specific response to the GP5 ectodomain from different PRRSV lineages after vaccination with commercially available modified live virus (MLV) vaccines. Methods: Post-weaning pigs were vaccinated with MLV vaccines derived from either lineage 1D (Prevacent PRRS^®^) or lineage 5 (Ingelvac PRRS^®^). Animals were challenged with a heterologous (lineage 1A) strain at 64 days post-vaccination (dpv). Blood samples were collected at various times post-vaccination and challenge. Kinetics of antibody response to different PRRSV antigens were monitored and virus neutralization against archetypal and contemporary strains belonging to lineage 5 and 1A were evaluated. In addition, antibody responses to peptides derived from the GP5 ectodomain of different viral lineages were assessed. Results: Our results showed that the GP5-specific antibody response observed between 18 and 35 dpv was delayed compared to responses to the viral nucleocapsid protein. The polyclonal antibody response in both vaccinated groups showed similar levels of binding to variant GP5 peptides from different sub-lineages. Notably, in both vaccinated groups, the antibody directed to a peptide representing the GP5 ectodomain of a lineage 1C strain (variant 1C.5) displayed a rise in titer at 64 dpv, which was further increased by the challenge with the lineage 1A strain. Less than 50% of animals developed heterologous neutralizing antibodies post-vaccination with both MLV vaccines. However, higher neutralization titers were observed in all vaccinated animal post-challenge. Conclusions: Together, these data provide insights into the antibody responses to the GP5 ectodomain in MLV-vaccinated swine herds.

## 1. Introduction

Porcine reproductive and respiratory syndrome (PRRS) is one of the most contagious and economically significant diseases spreading amongst domesticated swine herds. PRRS has seen its estimated annual economic impact in the U.S. increase from over USD 660 million to approximately USD 1.2 billion in less than a decade [1,2]. The causative agent of PRRS, porcine reproductive and respiratory syndrome virus (PRRSV), is classified into two genotypes, type-1 (European type) and type-2 (North American type), sharing approximately 65% similarity at the nucleotide level [3,4]. PRRSV type-2, the dominant genotype circulating in the U.S. swine herds, is known for its ability to evolve, generating novel viral variants that spread through the swine population [5]. Several methods have been used to classify the type-2 variants that arise in swine population but the currently used schema for characterizing variants adopt phylogenetic variations in the *orf5* gene to describe nine major lineages that have evolved over time and the current dominant lineage 1 variants that have eight sub-lineages within lineage 1 [6,7]. It has been proposed that these viral lineages have evolved under pressure of vaccines currently used in the field, notably due to advantages incurred by their ability to evade immune protection in the host [6,8].

Several types of modified live virus (MLV) vaccines are available and represent different viral lineages [9]. However, given that PRRSV displays a wide range of genomic heterogeneity, the protection provided by MLV vaccines against clinical disease caused by newly emerging PRRSV variants has been inconsistent over the past decades [10,11,12,13]. Vaccination has been shown to reduce clinical signs resulting from infection with heterologous strains but does not completely prevent shedding or transmission of the virus [14]. These observations highlight the need to develop effective prophylactic methodologies, including designing a PRRSV vaccine with broad cross-protection to control the spread of this disease in swine herds.

Neutralizing antibodies (NAbs) block viral entry into the host cells by hindering viral binding to host receptors. Protection against PRRSV infection in pigs is strongly associated with the levels of NAbs generated by a previous exposure to the virus [11,15]. The viral glycoprotein 5 (GP5) encoded by *orf5* is a major component of the virus attachment complex that mediates viral attachment to the host cells [16]. GP5 harbors several B-cell epitopes targeted by antibodies, including a conserved neutralizing epitope involved in virus neutralization [17]. The antibodies generated against GP5 has been shown to be cross-reactive to different PRRSV type-2 isolates, possibly due to the presence of the conserved neutralizing epitope flanked by two hypervariable regions at the GP5 ectodomain [18]. During the immune response to PRRSV, a delayed onset of NAbs production, which starts at around 28 days post-infection (dpi), has been observed in vaccinated pigs [19]. In addition, GP5-specific antibodies were found in serum samples at the same time as NAbs [19]. However, total PRRSV-specific antibody responses could be detected as early as 7 dpi [20], indicative of an early non-neutralizing antibody response to the virus. Interestingly, several positively selected amino acids (aa) were identified surrounding the principal neutralizing epitope within the ectodomain of GP5 by sequence analysis [8,21]. Key variations of consensus amino acid sequences for each sub-lineage have been linked to antigenic sites on the GP5 ectodomain [6], indicating that the emergence of novel PRRSV variants may be associated with immune pressure on these sites. Altogether, these suggest that the GP5 ectodomain may have a major role in inducing protective immune responses. However, little is known about the kinetics of the host response to different GP5 ectodomains in immunized animals to inform on its role in providing protection.

In this present study, the kinetics of humoral responses to homologous and heterologous GP5 ectodomains upon vaccination with two commercial MLV vaccines and challenge with a lineage 1A PRRSV strain are described. Outcomes of this study would provide more insights to improve the efficacy of vaccines to combat PRRSV.

## 2. Materials and Methods

### 2.1. Peptides, Recombinant Proteins, Cells, and Viruses

Recombinant nucleocapsid (N) and GP5 5′ total (52 amino acids external to the viral envelope) were derived from PRRSV type-2 prototype VR-2332 belonging to lineage 5 as described previously [22]. Peptides, as listed in Table 1, were synthesized with >85% purity by Biomatik Corporation (Kitchener, ON, Canada). Type-2 PRRSV isolates IA/2014 (lineage 1A; GenBank accession number MZ423533) and D11-052871 (lineage 5) were grown in MARC-145 cells (ATCC: CRL-12231), while isolate 46/2020 (lineage 1C.5; GenBank accession number MZ423535) was grown in PAM-KNU (Cat. No. T0741, Applied Biological Materials Inc., Richmond, BC, Canada). Viruses were then titrated in MARC-145 cells using the Reed-Muench method [23]. The D11-052871 isolate, sequenced using Oxford Nanopore Technology chemistry [24], is nearly 100% identical to VR-2332 in the GP5 ectodomain, with a single D34G mutation in this region distinguishing D11-052871 from VR-2332. All viruses in this study were sequenced before use to ensure the genetic make-up of virus preparations.

### 2.2. Animals

**Figure 1:**
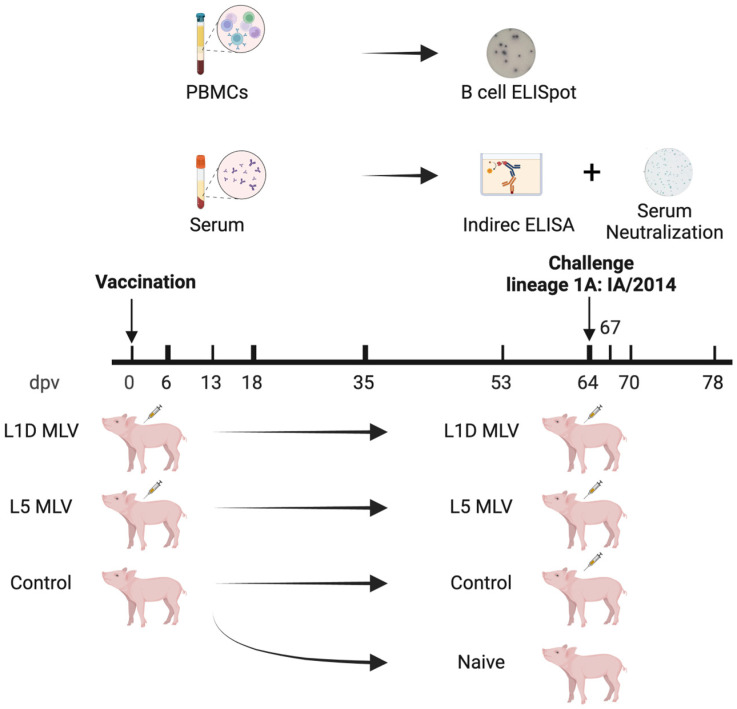
Timeline and experimental design. Pigs were either vaccinated with the respective modified live virus (MLV) vaccines or left unvaccinated, followed by a virus challenge (IA/2014) at 64 days post-vaccination (dpv). Thick ticks represent sample collections for both peripheral blood mononuclear cells and serum while the thin ticks represent sample collections for serum only. This figure was created with BioRender.com.

As shown in Figure 1, post-weaning female pigs (four-week-old) were divided into three groups randomly (*n* = 7 per group). These animals were obtained from a PRRS-negative herd that met the study criteria for pathogen-free status, defined as the absence of PRRSV, influenza virus, *Mycoplasma* species, and other respiratory pathogens. Two groups of pigs were vaccinated with either the Prevacent PRRS^®^ (lineage 1D) or Ingelvac PRRS^®^ (lineage 5) MLV vaccine, respectively, following the manufacturer’s instruction. A control group was kept unvaccinated. These animals were housed then in separate rooms in a BSL-2 facility with negative air pressure. Blood samples were collected at 0, 6, 13, 18, 35, 53, and 64 days post-vaccination (dpv). All vaccinated animals, as well as five pigs from the control group, were challenged with a lineage 1A virus (isolate IA/2014) at 64 dpv. The remaining two pigs from the control group were not challenged and were designated as the naïve group. For the challenge, all pigs were intramuscularly inoculated with 1 mL of the IA/2014 isolate at a dose of 10^5^ TCID_50_/mL. Fourteen days after the challenge, all pigs were euthanized for further analysis.

### 2.3. Enzyme-Linked Immunosorbent Assay (ELISA)

Venous blood was drawn using Vacutainer serum tubes (BD, Franklin Lakes, NJ, USA). Sera were separated from clotted blood samples by centrifugation at 1000× *g*, 4 °C for 30 min. The kinetics of antigen-specific antibody response were determined by ELISA. Briefly, antigens were diluted with carbonate bicarbonate buffer (50 mM, pH 9.6, ddH_2_O) to a desired concentration (1 µg/mL for N, GP5 5′ total and 0.5 µg/mL for the whole virus). The diluted antigens were coated onto each well in duplicate at 37 °C for 1 h and then the plates were washed with PBST (0.05% Tween-20, PBS) five times. Following that, plates were blocked with a blocking buffer (PBS, 1% BSA, 1% goat serum) at 37 °C for another 1 h. Serum diluted at different ratios with blocking buffer was then added to the plates, followed by incubation for 1 h at 37 °C before washing for five times. The HRP-conjugated goat anti-pig IgG Fc (1:50,000; Thermo Fisher Scientific, Waltham, MA, USA) was added to the plates and incubated at 37 °C for 30 min. Plates were washed five times and developed by adding 1-Step™ TMB ELISA substrate solution (Thermo Fisher Scientific). After incubation for 15 min without light, the reaction was stopped by adding 100 µL HCl (2N) and absorbance at 450 nm was measured using a microplate reader (BioTek, Winooski, VT, USA). The relative level of each sample was calculated by normalizing its absorbance at 450 nm (OD450) to the OD450 value of serum collected from a PRRSV-positive pig, which served as the positive control.

### 2.4. Microplate-Based Focus Reduction Neutralizing Assay

We performed a microplate-based focus reduction neutralization assay to assess the presence of neutralizing antibodies. Serum samples were first heat-inactivated at 56 °C for 30 min and then serially diluted two-fold in a 96-well plate. Heat-inactivated serum samples and minimum essential medium containing 2 × 10^5^ TCID_50_/mL of either D11-052871 or IA/2014 of PRRSV were mixed in an equal ratio and incubated at 37 °C for 1 h. Viruses incubated with 1% PRRSV-negative porcine serum collected from PRRSV-naïve animals served as the virus-only control, while the maintenance media served as the no-virus control. Then, 50 µL of the mixture was added to MARC-145 monolayers and incubated at 37 °C for 1 h. After that, the inoculums were removed, and fresh maintenance media were added. Following incubation at 37 °C for 11 h, the cells were fixed using 4% paraformaldehyde solution and permeabilized using 0.2% Triton X-100. The cells were then incubated with mouse monoclonal antibodies to PRRSV N protein (SR-30A, 1:10,000, 1 h at 37 °C; RTI, Brookings, SD, USA) and alkaline phosphatase-conjugated goat anti-mouse IgG (H+L) antibodies (1:1000, 40 min at 37 °C; Thermo Fisher Scientific) as the primary and secondary antibodies, respectively. The color was developed using 1-Step™ NBT/BCIP substrate solution (Thermo Fisher Scientific) at room temperature for 20 min before rinsing with water. The number of infected cells was then counted, and the neutralizing titer was defined as the highest serum dilution that resulted in a >50% reduction in the number of virus foci.

### 2.5. B-Cell Enzyme-Linked Immunosorbent Spot Assay

Venous blood was drawn from each animal in Vacutainer EDTA tubes (BD). The uncoagulated blood was first diluted 1:1 in PBS, layered on a lymphocyte separation medium (Corning, New York, NY, USA), and centrifuged for 30 min (400× *g*, room temperature). Peripheral blood mononuclear cells (PBMCs) were isolated from the interface and contaminating erythrocytes were lysed with ACK lysing buffer (Gibco, Grand Island, NY, USA). PBMCs were then washed with ice-cold PBS twice by centrifugation at 500× *g*, 4 °C for 15 min and resuspended in RPMI-1640 medium supplemented with 10% FBS, 20 mM HEPES, 1 mM sodium pyruvate, 1× non-essential aa, 100 U/mL penicillin, and 100 μg/mL streptomycin. MultiScreen-IP filter plates (Millipore, Burlington, MA, USA) were coated with different antigens (D11-052871, N, GP5 5′ total; 500 ng per well) before being blocked with the blocking buffer (RPMI-1640, 10% FBS). After counting, cells were seeded onto the blocked plates at a density of 10^6^ cells per well. Meanwhile, a mixture of recombinant human BAFF (50 μg/mL, PeproTech, Cranbury, NJ, USA), recombinant human soluble CD40 ligand (1 μg/mL, PeproTech), swine IL-21 (50 ng/mL, Kingfisher Biotech, Saint Paul, MN, USA), and 2-mercaptoethanol (50 µM) were added to the cells. The plates were washed with PBST five times after incubation for 24 h, and then incubated with alkaline phosphatase-conjugated goat anti-swine IgG (gamma) antibodies (1: 500; 1 h at 37 °C; KPL, Gaithersburg, MD, USA). Subsequently, plates were developed with 1-Step™ NBT/BCIP substrate solution at room temperature for 25 min and washed with PBST five times. After rinsing with tap water five times, the plates were stored in the dark and air-dried. Images of each well were then captured by an ImmunoSpot^®^ reader (CTL, Shaker Heights, OH, USA), and spots were counted manually. The number of spots indicated frequencies of cells actively producing a specific antibody in response to the corresponding antigen coated on the assay plate.

### 2.6. Statistics

Data were analyzed with GraphPad Prism 9 (GraphPad Software, La Jolla, CA, USA) using one-way or two-way ANOVA. Data are shown as mean ± SEM. * *p* < 0.05, ** *p* < 0.01, *** *p* < 0.001, **** *p* < 0.0001, ns = not significant.

## 3. Results

### 3.1. Vaccinated Groups Had Similar Antibody Responses to Different Viral Lineages

**Figure 2:**
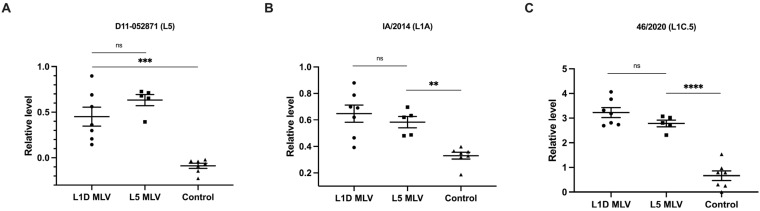
Relative levels of antibody responses in animals vaccinated with L1D or L5 vaccines, and controls, against PRRSV isolates from different lineages. Serum samples were collected at 64 days post-vaccination. (**A**) D11-052871 is a viral isolate belonging to lineage 5, (**B**) IA/2014 belongs to lineage 1A and (**C**) 46/2020 belongs to lineage 1C.5. Data were presented as mean ± SEM with individual values denoted by closed symbols. All optical density (OD450) values were normalized relative to a reference serum recognizing the corresponding antigen. ns = not significant; ** *p* < 0.01, *** *p* < 0.001, **** *p* < 0.0001, one-way ANOVA.

To determine if vaccine-induced antibodies recognized viruses from different lineages/sub-lineages, viruses belonging to lineage (L5) and 1 (L1A and L1C) were compared by ELISA using sera collected at 64 dpv. As shown in Figure 2, the magnitude of IgG antibody response to the whole virus belonging to different lineages/sub-lineages was not significantly different between vaccinated groups, indicating that both vaccines induced antibodies which recognized all lineages tested in the experiment. As expected, this PRRS virus-specific response was significantly greater in vaccinated animals than unvaccinated animals.

### 3.2. GP5-Specific Antibody Response Occurred at a Late-Stage Post-Vaccination

**Figure 3:**
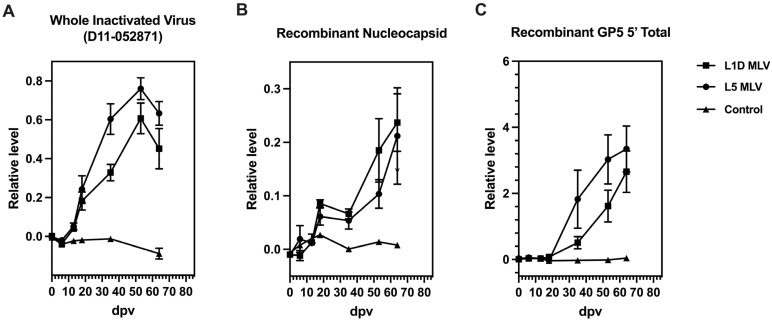
Kinetics of antibody responses to PRRSV antigens post-vaccination with L1D and L5 MLV. Serum samples were collected at 0, 6, 13, 18, 35, 53, and 64 days post-vaccination. (**A**) Kinetics of the L5 whole virus-specific antibody response post-vaccination. (**B**) Kinetics of the nucleocapsid (N)-specific antibody response post-vaccination. (**C**) Kinetics of recombinant GP5-specific antibody responses post-vaccination. Data are shown as means ± SEMs. Values were normalized as relative levels compared to a reference serum recognizing the corresponding antigen.

To determine the temporal nature of GP5 specific responses generated in the host post-vaccination, kinetics of antibody response to specific viral antigens were measured using serum collected at different time points post-vaccination. As indicated in Figure 3A, levels of antibody to the whole virus belonging to lineage 5 (D11-052871) increased after 6 dpv in both vaccinated groups and peaked at 53 dpv. The antibody to recombinant N increased between 18 and 35 dpv, earlier than the response to GP5 (Figure 3B,C). The whole virus antigen, recombinant N, and GP5 were all derived from a lineage 5 virus.

### 3.3. Homologous Response of GP5-Specific Antibody-Secreting Cells

**Figure 4:**
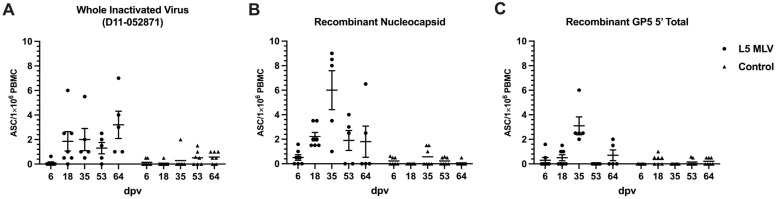
Frequencies of PRRSV antigen-specific antibody-secreting cells (ASCs) post-vaccination. PBMCs were isolated from unvaccinated animals or animals vaccinated with L5-derived MLV vaccine at different time points post-vaccination and then cultured to determine IgG-ASCs for different antigens including PRRSV D11-052871 (lineage 5) (**A**), N (**B**), and GP5 (**C**) using the ELISPOT assay. Data are shown as means ± SEMs.

Frequencies of antigen-specific antibody-secreting cells (ASCs) in circulation were measured using PBMCs collected at different time points post-vaccination. As shown in Figure 4, frequencies of circulating ASCs specific to the whole virus (L5) and N protein starts to increase earlier than those to GP5, which is consistent with the pattern of antibody responses seen in the serum (Figure 3). Viral antigen-specific ASCs in circulation peaked at 35 dpv and returned to baseline by 64 dpv.

### 3.4. Antibody Response and Amnestic Reponse to GP5 Ectodomain Peptide (aa 32–aa 61) Representing Different Viral Lineages

**Figure 5:**
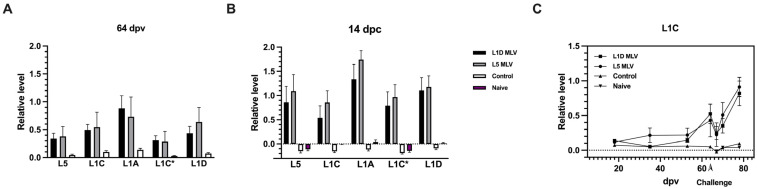
Antibody responses to peptides (aa 32–aa 61) designed from the GP5 ectodomain of different lineages. Relative levels of antibody response to the GP5 ectodomain are shown using serum samples collected at (**A**) 64 dpv and (**B**) 14 days post-challenge (dpc) from vaccinated and challenged (L1D MLV and L5 MLV), unvaccinated and challenged (control), or unvaccinated (naïve) groups. Peptides corresponding to L5 (D11-052871), L1C (46/2020), L1A (IA/2014), L1C* (KP283416) and L1D (KY348849) were used to assess vaccination response. Two-way ANOVA. (**C**) Kinetics of antibody response to the GP5 ectodomain peptide derived from 46/2020 (lineage 1C.5). The arrow indicates challenge with an L1A isolate (IA/2014) at 64 dpv. Data are shown as means ± SEMs. Values are normalized as relative levels compared to a reference serum.

To determine whether vaccines elicited antibodies to the GP5 ectodomain, response to synthetic peptides encompassing the GP5 ectodomain derived from different lineages/sub-lineages (sequence shown in Table 1) were evaluated using sera collected at 64 dpv and at 14 days post-challenge (dpc). As indicated in Figure 5A,B, no statistical difference was observed between the two vaccinated groups in the magnitude of responses to the GP5 ectodomain from various viral lineages post-vaccination and post-challenge, albeit they were higher than responses observed in the unvaccinated, challenged (control) and unvaccinated, unchallenged animals (naïve). The kinetics of antibody responses to the GP5 ectodomain (of L1C) demonstrate that the GP-5 specific antibody production increases only at 35 dpv. However, when vaccinated animals were challenged with a heterologous virus (compared to the vaccine), a distinct anamnestic response to the GP5 peptide was observed within the first week post-challenge in both vaccinated groups. This response was not induced in either the control or naïve group.

### 3.5. Increased Serum Neutralization Activity Post-Challenge

To determine the effectiveness of the vaccine response to neutralize virus infection, neutralizing titers of serum collected at 64 dpv and 14 dpc were assessed against two viruses belonging to the lineage 5 and 1A. A microplate-based focus reduction neutralizing assay was employed. As shown in Table 2, less than half of animals had neutralizing titers > 8 at 64 dpv, which was just before the challenge. However, about 60% of pigs vaccinated with the L5 MLV generated neutralizing titers > 8, when assessed against the homologous strain. Interestingly, neutralizing titers against the L5 virus were higher regardless of whether the pigs had received the L5 or L1D vaccine. After the challenge, all vaccinated animals developed increased NAbs against both homologous and heterologous strains. These findings suggest that, with pre-existing vaccine-induced immunity, animals are likely to produce broadly NAbs against PRRSV following natural infection or live virus inoculation.

## 4. Discussion

The first MLV vaccine derived from a lineage 5 isolate was made commercially available in the U.S. in 1994 [25], and several additional vaccines representing multiple lineages have been developed and marketed since then. Although these MLV vaccines have achieved partial protection against homologous strains, their effectiveness against heterologous strains remains limited. With the evolutionary landscape of PRRSV strains continuously expanding over the last several decades, developing a broadly cross-protective vaccine remains an urgent priority for the successful control of PRRS outbreaks [26]. The two MLV vaccines evaluated in the present study induced virus-specific antibodies that recognized viruses of different lineages and sub-lineages (Figure 2), indicating some degree of cross-reactivity of antisera generated in vaccinated animals, regardless of the viral lineage included in the vaccines. Our findings are consistent with previous studies, which described antisera collected from infected pigs were cross-reactive using ELISA or immunofluorescent assay [27,28,29]. However, it is well known that antibody binding responses do not accurately reflect the ability of the host to cross-protect against heterologous virus strains [20]. It should be noted that PRRSV infection can induce a significant expansion of B-cell populations, resulting in the production of large quantities of antibodies that lack virus-neutralizing capabilities, a phenomenon associated with polyclonal B-cell activation and the differentiation of B-cells into immunoglobulin-producing cells driven by non-specific helper T-cell responses [30]. Therefore, further investigations on the production and function of antibodies to specific viral antigens are of great importance to better understand PRRSV-induced humoral responses and cross-protection.

Kinetics of antibody responses to different viral proteins have been described in several previous studies. Our findings indicate that antibodies against the whole viral particle were undetectable until about a week after exposure (Figure 3A), while N protein-specific antibodies began increasing around the same time (Figure 3B). Consistent with these results, previous studies have reported N protein-specific antibodies were detectable as early as one-week post-infection and persist up to five months [31,32,33]. In contrast, GP5-specific antibodies appeared later, between 18 and 35 dpv (Figure 3C), aligning with studies that identified GP5-specific responses in serum samples collected four weeks post-infection using immunoblotting and ELISA [19,32,33]. Due to their early appearance and prolonged persistence, N protein-targeting antibodies are commonly used to evaluate PRRSV seroconversion, with most commercial ELISA kits designed to detect this antigen [34]. However, studies focusing on the kinetics of PRRSV-specific antibody production often rely on these kits, which may overlook the temporal separation of responses to different viral antigens.

A similar temporal pattern of GP5-specific ASCs in the blood was found in our studies (Figure 4), confirming the delayed recognition of this viral antigen compared to ASCs specific to the N protein. The dynamic shift in antibody-antigen recognition during the host response to PRRSV infection suggests that the process of viral recognition, whether due to infection or vaccination, may contribute to the delay in the development of neutralizing antibodies in swine [35]. The development of GP5 antibodies observed in our studies attests that the primary neutralizing epitope on the GP5 ectodomain may not be recognized by the host until later post-vaccination. The immunological mechanism for this lack of or delay in recognition may be a function of antigen abundance of certain proteins like the viral nucleocapsid [36], which leads to a higher propensity of these proteins to be presented or recognized. On the other hand, the delay of antibody production may be also attributed to impaired antigen presentation due to the downregulation of swine leukocyte antigen class II resulting from PRRSV infection [37]. Moreover, several studies have found that PRRSV MLV and wild-type strains induced massive apoptosis of T-cell precursors and altered the T-cell receptor repertoire in the thymus by depletion certain PRRSV-specific T-cells from the repertoire, which can further disrupt the immunological response of the animals and potentially explain the delayed response in the host.

The protective role of antibodies to GP5 has been controversial. Although Li and Murtaugh claimed that affinity-purified antibodies raised against the GP5 ectodomain from PRRSV antisera showed no neutralizing abilities [38], Young et al. showed that monoclonal antibodies to GP5 were able to neutralize homologous strains [18]. The incongruity in the neutralizing role of GP5-specific antibodies may be explained by the timing of serum collection in Li and Murtaugh, which was six weeks post-infection with VR-2332, a point when neutralizing antibodies generated against GP5 might not have reached protective levels [38]. On the other hand, Young et al. used monoclonal antibodies developed from hyperimmune sows given repeated exposures to PRRSV, which may foster the effective maturation of B-cell to produce GP5-directed neutralizing antibody [18].

The GP5 ectodomain consists of three unique antibody binding sites, with many studies demonstrating neutralization activity is concentrated on a conserved epitope (aa 37 to aa 45), also known as epitope B. Epitope B has been claimed as a primary recognition site for broadly neutralizing antibodies [17]. Mutations in epitope C (aa 51 to aa 57), found downstream from epitope B, have been shown to confer a survival advantage against host immune responses and, as a result, epitope C has been proposed as a target for homologous neutralization [39]. Another epitope upstream of epitope B was also identified and designated as epitope A (aa 27 to aa 31) [17]. Peptides derived from epitope A have been shown to bind to non-neutralizing serum but not to neutralizing serum [17]. Given that epitope A is known to be immunodominant but considered to be a non-neutralizing epitope [17], the present study focused on investigating the production of antibodies targeting the neutralizing B and C epitopes on the GP5 ectodomain. The results showed that the host generated antibodies against both epitope B and C on GP5 with similar binding to peptides from different viral lineages tested, both before and after challenge with a heterologous viral strain (Figure 5A,B). This observation is likely attributable to the conserved nature of epitope B, although the impact of epitope C specific antibodies could not be elucidated in this study. In addition, this study described the kinetics of antibody production against these the B/C epitopes (Figure 5C). GP5 epitope B/C antibody levels remained low until 53 dpv, delayed later than the rise of antibodies to the recombinant GP5, which includes the complete ectodomain of GP5. Kick et al. reported that MLV inoculated animals produced detectable NAbs against homologous strains within 35 dpi [40]. These results taken together suggest that antibodies generated against the GP5 ectodomain may play a significant role in virus neutralization. Also, within the first week after challenge, there was an increase in the levels of antibodies directed against the epitope B/C peptide (Figure 5C), which suggests an anamnestic B-cell response to previous exposure to the antigen. Viral challenge has been shown to increase neutralizing antibody (NAb) titers to heterologous strains in MLV-vaccinated animals [13,41], which is consistent with the results shown in Table 2. These findings further add that the Ab to the GP5 ectodomain may play a significant role in broad serum neutralization. It has been shown that epitope B specific antibodies titers are positively correlated to the NAb titer in animals [42]. Although not tested in this present study, the increased NAb titers post-challenge may be associated with the anamnestic response to epitope B, which is conserved across strains.

A prime–boost strategy has been proposed as an efficient way to induce a strong antibody response against a broad range of viral variants [43,44,45]. Re-exposure to a homologous or heterologous PRRSV strain has been shown to result in increased heterologous NAb titers [46,47,48]. Though the present study did not include a booster dose of the MLV vaccine, challenge with a heterologous L1A isolate elicited higher neutralizing antibody titers against both homologous and heterologous virus variants at 14 dpc, compared to pre-challenge sera at 64 dpv (Table 2). Although NAb titers were only measured against two variants in this study, the question surrounding whether antibodies elicited by this heterologous L1A challenge in MLV-vaccinated pigs are broadly neutralizing remains. It is worth noting that the increased NAb titers against heterologous strain might be due to memory responses directed to the conserved neutralizing epitope in GP5, given that neutralizing epitopes identified in other structural proteins including GP2, GP3, and GP4 have been shown to be variable between strains [49]. However, it cannot be ruled out that the enhanced neutralizing antibody response observed in this study may also target complex conformational epitopes, including those potentially involving interactions with the host receptor CD163, which may not be exclusive to the GP5 ectodomain [50]. Further investigations are required to identify the precise antigenic determinants involved in this response. The results also suggest that a single MLV vaccination dose establishes a foundational immune memory, which may not confer strong neutralizing protection initially, as observed two months post-vaccination. However, upon heterologous strain infection, the rapid rise in neutralizing antibodies within two weeks indicates a robust amnestic response against certain neutralizing epitopes including GP5 (Table 2). This swift immune enhancement, though not accompanied by recorded clinical symptom alleviation in this study, could suggest potential protective benefits in a field scenario. Vaccinated animals exposed to PRRSV, either through natural infection or subsequent vaccination, might experience a faster and stronger immune response, potentially mitigating clinical symptoms. While further studies are needed to confirm these clinical outcomes, this rapid immunological boost could have significant implications for optimizing future vaccination paradigms aimed at population-level protection with a multi-dose strategy [51]. In the meantime, different prime–boost vaccination regimens, including a MLV prime-subunit protein boost could be explored, to further identify specific viral antigens involved in broad viral neutralization. Given the temporal dynamics of antibody responses to specific viral proteins, an optimal time point for boosters should also be explored. The delay in eliciting GP5-specific antibodies coincides with a similar time frame for production of neutralizing antibodies [20], it would be prudent to allow at least four to six weeks post primary exposure to boost the vaccine response.

## 5. Conclusions

In conclusion, this present study characterized a delayed GP5-specific humoral response in MLV-vaccinated animals. Meanwhile, limited NAbs were elicited by a single dose of MLV vaccines, but the heterologous challenge led to enhanced serum neutralizations of homologous and heterologous strains, indicating that a prime–boost approach may endow pigs with better protection against PRRSV.

## Figures and Tables

**Table 1:** Amino acid sequence for synthesized peptides derived from GP5 ectodomain (amino acid 32 to 61) of different PRRSV type-2 lineages/sub-lineages used in this study.

Isolate ID	Sequence (N to C Terminus)	Lineage
D11-052871	SNGSSSHLQLIYNLTLCELNGTDWLANKFD	L5
46/2020	NNSSSSHLQLIYNLTICELNGTDWLNERFD	L1C
IA/2014	NNSSSSHLQLIYNLTICELNGTDWLNKTFD	L1A
KP283416	NSNSSSHLQLIYNLTICELNGTDWLSKKFD	L1C *
KY348849	SSNSSSHLQLIYNLTICELNGTDWLNNEFD	L1D

* Another lineage 1C variant other than 46/2020.

**Table 2:** Virus neutralization using serum samples collected from the L1D and L5 at 64 dpv and 14 dpc. Titers greater than eight are considered protective.

Group/Isolate	Pig ID	64 dpv		14 dpc	
		IA/2014 (L1D)	D11-052871 (L5)	IA/2014 (L1D)	D11-052871 (L5)
L1D MLV	1–5	<4	4	>128	32
	1–7	<4	64	64	>128
	1–9	<4	32	>128	>128
	1–13	8	64	>128	>128
	1–14	<4	<4	32	32
	1–15	32	<4	>128	>128
	1–21	4	4	64	>128
Neutralization (%)		2/7 (28.6%)	3/7 (42.9%)	7/7 (100%)	7/7 (100%)
L5 MLV	2–3	<4	8	>128	>128
	2–4	<4	16	64	>128
	2–8	32	32	>128	>128
	2–12	<4	<4	>128	>128
	2–20	<4	4	>128	>128
Neutralization (%)		1/5 (20.0%)	3/5 (60.0%)	5/5 (100%)	5/5 (100%)

## Data Availability

The data presented in this study will be available upon reasonable request from the corresponding author.

## Abbreviations

The following abbreviations are used in this manuscript:

PRRS	Porcine reproductive and respiratory syndrome
PRRSV	Porcine reproductive and respiratory syndrome virus
MLV	Modified live virus
NAbs	Neutralizing antibodies
GP5	Glycoprotein 5
N	Nucleocapsid
dpv	Days post-vaccination
dpi	Days post-infection
dpc	Days post-challenge
aa	Amino acids
PBMCs	Peripheral blood mononuclear cells
